# Practices and perspectives of patients and healthcare professionals on shared decision-making in nephrology

**DOI:** 10.1186/s12882-022-02887-4

**Published:** 2022-07-21

**Authors:** Sandra van Dulmen, Ruud Roodbeen, Lotte Schulze, Karen Prantl, Maarten Rookmaaker, Brigit van Jaarsveld, Janneke Noordman, Alferso Abrahams

**Affiliations:** 1 Nivel (Netherlands institute for health services research), PO Box 1568, 3500 Utrecht, BN Netherlands; 2 Department of Primary and Community Care, Radboud university medical center, Radboud Institute for Health Sciences, Nijmegen, Netherlands; 3 Faculty of Caring Science, Working Life and Social Welfare, University of Borås, Borås, Sweden; 4 Breuer&Intraval, Research and Consultancy, Groningen, Netherlands; 5 Dutch Kidney Patients Association, Bussum, Netherlands; 6 Department of Nephrology and Hypertension, University Medical Center Utrecht, Utrecht, Netherlands; 7 Department of NephrologyAmsterdam UMC, Vrije Universiteit Amsterdam location, Amsterdam, Netherlands; 8 Diapriva Dialysis Center, Amsterdam, Netherlands

**Keywords:** Communication, Shared decision-making, Nephrology, Observational study, Video-recording, Stimulated recall interviews, Qualitative study

## Abstract

**Background:**

Given the complexity and variety in treatment options for advanced chronic kidney disease (CKD), shared decision-making (SDM) can be a challenge. SDM is needed for making decisions that best suit patients’ needs and their medical and living situations. SDM might be experienced differently by different stakeholders. This study aimed to explore clinical practice and perspectives on SDM in nephrology from three angles: observers, patients and healthcare professionals (HCPs).

**Methods:**

An explanatory sequential mixed methods design was used. First, in the quantitative part of the study, outpatient consultations with patients with advanced chronic kidney disease (eGFR < 20 ml/min) were video recorded and SDM was assessed using the OPTION^5^ instrument. Subsequently, in the qualitative part, patients and HCPs reflected on their own SDM behaviour during individual stimulated recall interviews which were analysed using deductive thematic content analysis.

**Results:**

Twenty nine consultations were recorded and observed in seven hospitals. The mean SDM score was 51 (range 25–80), indicating that SDM was applied to a moderate extent. The stimulated recall interviews with patients showed that they rely on the information provision and opinion of HCPs, expect consistency and support, and desire a proactive role. They also expect to be questioned by the HCP about their SDM preferences. HCPs said they were willing to incorporate patients’ preferences in SDM, as long as there are no medical contraindications. They also prefer patients to take a prominent role in SDM. HCPs ascribe various roles to themselves in supporting patients’ decision-making.

**Conclusions:**

Although SDM was applied by HCPs to a moderate extent, improvement is needed, especially in helping patients get the information they need and in making sure that every patient is involved in SDM. This is even more important given the complex nature of the disease and the relatively high prevalence of limited health literacy among patients with chronic kidney disease.

**Supplementary Information:**

The online version contains supplementary material available at 10.1186/s12882-022-02887-4.

## Background

Patients with advanced chronic kidney disease (CKD) need to choose a kidney replacement therapy that best suits their needs, medical options and living situation [1]. In 2018, at least 17,670 Dutch patients received kidney replacement therapy [2]. Although various forms of dialysis have comparable clinical outcomes, the treatment options are preference-sensitive and impact patients’ life differently [3–5]. This leaves both patients and healthcare professionals in nephrology (HCPs) with the task of making a treatment decision together. To support this process, Dutch patients with CKD receive treatment modality education.

To make a decision, HCPs are legally required to fully inform patients about all available options, risks and consequences, apprise themselves of the patients’ situation and personal circumstances, and invite them to ask questions [6]. The Dutch Medical Treatment Act also obliges HCPs to make a treatment decision together with the patient [7, 8]. In such a process of shared decision-making (SDM), HCPs and patients choose the best evidence-based treatment option together after discussing all available options, their pros and cons, personal preferences, and the circumstances of the patient [7–9]. In other words, SDM results from weighing clinical guidelines against patient preferences.

Given the complexity of CKD and the preference-sensitive choice to be made, SDM in nephrology is challenging [10]. Our recent study shows that, notwithstanding best intentions, nephrologists tend to steer the decision-making process in a certain direction, thereby leaving less room for SDM [11]. Additionally, even after adjusting for the patient’s age, sex and disease stage, Dutch dialysis centres vary widely in the types of treatments provided, especially in the percentage of home dialyses out of the total number of dialysis treatments [12]. This suggests that SDM in Dutch nephrology is not yet widely implemented.

To our knowledge, no studies have so far investigated SDM during consultations in nephrology. Our first aim was therefore to assess SDM during consultations in which a decision for kidney replacement therapy was discussed. Our second aim was to have patients and HCPs reflect on their own communication and SDM behaviour for additional in-depth understanding of the practice of SDM in nephrology.

## Methods

### Study design

This study is embedded within a larger Dutch nephrology study called DIALOOG. Twenty-eight HCPs (i.e. nephrologists) from 21 Dutch hospitals were invited to participate (invited by AA and BvJ). In three academic and four general hospitals, data was collected between July 2019 and November 2020 by two researchers (RR and LS). Fourteen nephrologists and one specialist nephrology nurse who discussed final treatment options with patients with advanced CKD participated.

An explanatory sequential mixed method design was used with a quantitative observational part and a qualitative interview part [13]. The quantitative part started by collecting video-recorded outpatient consultations with the aim of 1) observing the extent to which SDM was applied in clinical practice, and 2) providing the basis for the stimulated recall interviews with patients and HCPs. Video recordings of consultations are a valid method for examining communication, a video recorder does not significantly influence behaviour [14–16]. SDM was investigated by the extent to which HCPs involve patients in SDM, using the ‘Observing Patient Involvement in Decision-Making’ (OPTION)^5^ instrument [17–19]. This instrument is used for analysing the video-recorded consultations by coding five items (see Table 2) for every individual video recording. This coding assesses the extent to which HCPs involve patients in SDM.

The theoretical framework underpinning the qualitative part of this study is based on a phenomenological approach, focusing on describing the meaning and significance of experiences. This design lets participants express their views on communication and SDM in nephrology in their own words. In the qualitative interview part, patients and HCPs reflected on their own SDM behaviour during individual stimulated recall interviews together with a researcher. During these interviews, video-recorded consultations were used to recall the outpatient consultation and to discuss patients’ and HCPs’ elicited perspectives, thoughts and reactions [20–23]. The participants were told that nothing they said would be passed on to others.

### Procedure

Patients with CKD (eGFR < 20 ml/min) were invited to participate in the study by the participating HCPs, based on inclusion criteria and convenience sampling. They had to be aged =18 and awaiting a final consultation with their HCP to discuss a form of kidney replacement treatment. Patients were excluded from participating if they 1) were not able to speak Dutch sufficiently well, 2) had a severe intellectual disability, or 3) had a psychiatric problem or dementia. These consultations mark the end of a multi-consultation, educational process and aim to summarize all treatment aspects discussed previously.

### Recruitment

We used the following procedure, which is the same as described in our previous paper reporting results of another analysis of the same dataset [11]. The HCPs of the participating hospitals were the contacts for the researchers. After local approval on feasibility from the participating hospital and selecting and inviting patients by the HCPs, the HCPs and/or researcher phoned eligible patients about 1 week before the scheduled visit to the hospital. During this phone call, the patients were informed in more detail about the aim and procedure of the study. The patients who agreed to participate were asked to sign an informed consent (IC) form before seeing their HCP. The HCPs signed an IC form too. For patients who have difficulty reading and understanding health-related information, plain language versions of the information letter and the IC form were made [11]. Before seeing the HCP, patients filled out a questionnaire with questions about their sociodemographic characteristics and their preference for participating in treatment decision-making. The outpatient consultation was then recorded using an unmanned camera. During the recording, only the HCP was visible. Afterwards, the video recordings were coded in a secured, locked room at Nivel [11].

### Quantitative video-observations

OPTION^5^ is a reliable and valid instrument for investigating SDM [17–19]. Using OPTION on every video recording allows the extent to which HCPs involve patients in SDM to be assessed. Five SDM items (Table 2) are coded on a 5-point Likert scale, (0 = ‘zero effort observed’ to 4 = ‘exemplary effort’). The total OPTION^5^ score is generated by converting the scores to a 0–100 scale and then calculating the average. The higher the score, the higher the level of SDM. All 29 video-recorded consultations were coded by the main observer (RR), and four consultations were also coded by a second observer (JN). These four consultations were randomly selected. A rule of thumb is to have at least 10% of records independently coded by two observers [24, 25]. The coding results of the main coder (RR) are presented in Table 2. Inter-rater reliability between observers was calculated using Cohen’s kappa (.412), indicating a moderate inter-rater agreement [26, 27].

### Qualitative stimulated recall interviews

After the video-recorded consultation, individual patients and HCPs looked back at carefully selected fragments of their own consultations with a researcher (RR, male, or LS, female). Recall interviews with patients were conducted at home or another place convenient for the patient (e.g. at the hospital in a private room), with the researcher, the patient, and in some cases, the significant other of the patient present. Recall interviews with HCPs were conducted at their workplace, with only the researcher and HCPs present. Only the researcher and the participants were present. Both researchers were occupied as communication researchers (MSc) at the time of the study, and as such, both investigated (and had general interests in) communication in multiple domains and settings in healthcare (e.g. in palliative care, primary care, and nephrology for DIALOOG). The researchers were experienced in conducting qualitative in-depth semi-structured interviews and stimulated recall interviews with patients and HCPs to reflect on their own SDM and communication behaviour (see e.g. 32). Both researchers were trained by co-authors (SvD and JN) to conduct interviews with HCP and patients, and both had no profound prior relationship with the participants. Furthermore, the participants were aware that the researchers were not medically trained or involved in patient care, and participants generally knew the goals and reasons behind the interviews (i.e. investigating clinical practice and perspectives on communication in nephrology).

Characteristics of the interviewed patients (age, sex and presence of significant other) and HCPs’ interviews (number of consultations discussed) were noted and so was the duration of the interviews. All interviews were audio-recorded, transcribed verbatim and anonymized; no field notes were made. Transcripts were not returned to participants for comments or corrections. During the interviews, patients and HCPs reflected independently on their own communication and SDM, and on the communication and SDM of the other person in the video-recorded consultations [28]. To do so, a topic list was developed (Appendices 1 and 2) based on literature [29], experience from previous research (RR, LS, JN and SvD) and feedback from two nephrologists (AA and BvJ). The list was not pilot-tested. For each interview, three fragments were selected to show to the patient as well as to the HCP. These fragments were approximately 2 min in length, based on the occurrence of SDM (using the OPTION^5^ protocol). The average duration of the interviews with patients, per consultation, was 54 minutes (range between 26 and 104 minutes). The average duration of interviews with HCPs was 37 minutes (range between 23 and 67 minutes).

### Analysis

The interview transcripts were analysed using deductive thematic content analysis [30]. Deductive means that the themes presented in the results section are similar to the questions in the topic lists (see Appendices 1 and 2). Thematic content analysis indicates the process of reading, coding, comparing and discussing the answers provided by patients and HCPs and finalizing these answers to give presentable results. Results from patient interviews could be categorized into three main questions: 1) what is important in SDM for you, 2) what can you do better, and 3) what can your HCP do better? For the HCPs, these questions were 1) what is important in SDM for you, 2) what contextual factors could have influenced SDM, 3) what role do you give yourself in SDM, and 4) what could you do better? All transcripts were read carefully by one coder (RR) and parts in which elements of SDM were mentioned were selected. An initial coding was applied to the selected segments, discrepancies between researchers were resolved through discussion by two additional coders (JN and SvD, with RR), and modifications to the initial categories were made where necessary. All categories and patterns that emerged during analysis are illustrated by quotes (Tables 3 and 4). Participants did not provide feedback on the findings.

## Results

### Sample characteristics

Twenty-nine patients and 15 HCPs participated (Table 1). The patients had a mean age of 71 and 14 were women. Consultations lasted around 30 minutes. Thirty consultations were video-recorded, of which one was excluded because the patient appeared to have an intellectual disability. Fourteen stimulated recall interviews were conducted with patients and 16 with HCPs.

**Table 1:** Characteristics of participating patients and professionals

**Video-recorded consultations (*n*** **= 29)**		
Duration (in minutes)	*Mean (SD)* ^**^	*Range*
	28:96 (12:42)	14:46–62:51
Type of consultations	*Number (%)*	
- New	5 (17)	
- Control (i.e. follow-up consultations)****	24 (83)	
**Characteristics of patients in video-recorded consultations (n = 29)**		
	*Mean (SD)*	*Range*
Age (in years)^***^	71.4 (10.5)	49–89
Sex	*Number (%)*	
- Male	15 (52)	
- Female	14 (48)	
**Characteristics of professionals in video-recorded consultations (*n*** **= 15)**		
	*Mean (SD)*	*Range*
Average number of consultations per professional	2.1 (1.3)	1–5
Sex	*Number (%)*	
- Male	6 (40)	
- Female	9 (60)	
Profession		
- Nephrologist ^#^	13 (87)	
- Specialist nephrologist nurse	2 (13)	
**Interviews with patients (interviews conducted;** ***n*** **= 14)**		
	*Mean (SD*	*Range*
Duration (in minutes)	54:23 (18:24)	25:55–103:36
Age (in years)	72.9 (8.1)	57–83
Sex	*Number (%)*	
- Male	8 (57)	
- Female	6 (43)	
Significant others present during interview	7 (50%)	
**Interviews with professionals (interviews conducted;** ***n*** **= 16**^**##**^**)**		
	*Mean (SD)*	*Range*
Duration (in minutes)	37:28 (10:55)	22:55–69:58
Number of consultations discussed (*n* = 21^##^) per professional	1.5 (0.7)	1–3

Notes

* OPTION5, observing patient involvement in decision making. ** SD = standard deviation. *** one missing

**** Control or follow-up consultations are consultations in which the patient and HCP have routine consultations with each other, and in addition to discussing a decision for kidney replacement therapy, discuss other routine information as well

# Two nephrologists were in training during the project

## We met two professionals twice to allow the interviews to be completed

### 3.2. Quantitative video observations

The mean SDM score (0–100 score) was 51 (SD = 14). The highest average score (see Table 2), was observed for items 1 and 4, the lowest for item 2. This indicates that, on average, the effort put into involving patients in SDM in practice was moderate (basic phrases or sentences used).

**Table 2:** OPTION^5^* scores from observations of patients and professionals (*n* = 29)

	0	1	2	3	4	Mean (SD)
**1.** For the health issue being discussed, the clinician draws attention to or confirms the fact that there are alternate treatment or management options or that a decision needs to be taken. If the patient rather than the clinician draws attention to the availability of options, the clinician responds by agreeing that the options need deliberation.	1	1	13	13	1	2.4 (0.8)
**2.** The clinician reassures the patient or reaffirms that they will support the patient in informing them or deliberating the options. If the patient states that they have sought or obtained information before the meeting, the clinician supports the deliberation process.	7	13	6	3	0	1.2 (0.9)
**3.** The clinician gives information or checks understanding about the options that are considered reasonable (this can include taking no action), to support the patient in comparing alternatives. If the patient requests clarification, the clinician supports the process.	0	3	18	4	4	2.3 (0.8)
**4.** The clinician makes an effort to elicit the patient’s preferences in response to the options that have been described. When the patient states their preference, the clinician is supportive.	2	4	6	14	3	2.4 (1.1)
**5.** The clinician makes an effort to integrate the patient’s elicited preferences as decisions are made. If the patient indicates how best to integrate their preferences as decisions are made, the clinician makes an effort to do so.	2	10	7	10	0	1.9 (1.0)
**Total**	**12**	**31**	**50**	**44**	**8**	**50.9 (14.4)**

Notes

*OPTION^5^, observing patient involvement in decision making

Score description

0 = No effort (zero effort observed).

1 = Minimal effort (effort to communicate could be implied or interpreted)

2 = Moderate effort (basic phrases or sentences used)

3 = Skilled effort (substantive phrases or sentences used)

4 = Exemplary effort (clear, accurate communication methods used)

### Qualitative stimulated recall interviews

The stimulated recall interviews took place between September 2019 and November 2020, conducted by two researchers (RR and LS) within, on average, 7 weeks after the recorded consultation. The patient interviews lasted 54 minutes on average and the HCP interviews 37 minutes (Table 1). Tables 3 and 4 show questions, themes and illustrative quotes regarding SDM perspectives of patients and HCPs respectively. In this section, the most salient examples of diverse perspectives are presented.

**Table 3:** Questions, themes and patients’ quotes

Themes	Quotes
**What is important in SDM for patients?**	
HCP’s opinion	I: And why do you think he is better able to decide? P: He knows everything about it [about dialysis], he knows the consequences, he knows the impact on your life. So, I am … I really like it when I ask for his advice, like, ‘“What would you advise, what do you think is the best thing for me to choose?” I like it when he answers honestly. P: Yeah, he’s the expert, and he knows. See, I only have information from stories and books. And he knows what it actually does to your life. So, I like that. Yes. *- Female, aged 64*
Medical options	P: I don’t know if … well, as a patient, you can of course have certain desires, but if that isn’t medically justified or possible, then that’s it, everything stops. And then there comes the input from the doctor, who says “Okay, that’s all well and good, but it probably won’t work for you”, or “We can start with that, but the question is whether we can keep it like that”. So there are other sides to the story too. I can have all those desires … I: Yes, and not everything is possible. P: Not everything is possible. *- Male, aged 76*
Consider the environment	I: And do you ask for the doctors’ advice and take that in mind? SO: Yes, I take it home and then we have a discussion at the table together. *- Female, aged 68*
Type of provision of information	SO: And we’ve visited people, through the arrangements of the hospital. At home with a woman, who has been doing this [PD dialysis] for 2.5 years. […]. P: Yes, and we were received very nicely by these people. Absolutely great. SO: That was a positive thing, of course … P: They showed us everything, how it [PD dialysis] works in everyday life, no secrets. Fantastic! I: Okay, well that’s very nice. P: And after that [after the visit], you look at it differently, you look at it very differently. *- Female, aged 74*
Timeline of SDM before starting treatment	SO: Yes, but you also thought it was a step further, that now you had to visit that vascular surgeon, that it [dialysis] is getting closer and closer. P: Yes, and you asked the doctor, “How long could it take if it stays that way?” Then she said, “It could take another year [before dialysis starts]”. If nothing gets in the way. SO: Yes, yes. It can take a year, but it can also be within a year [that dialysis starts]. They just can’t answer that. And they are very honest about that, I mean, if he gets sick [the patient], then dialysis could start right away. So they can’t say that [predict when dialysis will start] and they are honest about it. *- Male, aged 77*
**What can patients themselves do better in SDM?**	
Take control during the conversation	P: Maybe I would interrupt him in the 80+ story, huh. That I would say, “Man, you don’t have to explain all that to me, that’s irrelevant in my situation.” That is maybe what I should have done during the conversation. Because he talked about it quite extensively [about conservative therapy]. So maybe I should have said “Yeah, but that’s not the case at all, so tell me more about the difference between haemodialysis and abdominal dialysis instead.” Maybe I could have been clearer about that because it’s still unclear to me. *- Female, aged 64*
Ask (more) questions	P: Maybe, I would ask the doctor, “Okay, I’m too young [for conservative treatment]. But what is conservative treatment exactly, how does it work? What medication is required? What’s the prognosis? How many years can you live on it? At what stage? There’s so much I don’t know. Also, I don’t know what stage I’m at now, regarding my kidney function – I don’t know. I forgot to ask her that question because I don’t know. *- Female, aged 57*
Take notes	I: Because, how do you do this when you forget things and sit at home and think, “What about that?” SO: Yes, then we’ll ask these questions the next time. […] You don’t write anything down, and that’s actually a typical human error. I: That you think you’ll remember what has been said? SO: I have to write that down! Use a cheat sheet and take it everywhere with you when you go somewhere. Just like when going out for groceries. *- Female, aged 68*
**What can HCPs do better in SDM according to patients?**	
Be consistent and reliable when providing information	P: The information is correct, only I imagined when I got into this that I could only choose between three options. Namely doing nothing, home dialysis, or some other form of the two dialyses, abdominal dialysis, or in the hospital, right? But now he mentioned a transplant. But at the clinic, they told me transplantation was out of the question because I’ve turned 70 and that’s the limit. They said I didn’t stand a chance of getting a transplant. And now he suddenly provides me this option! *- Male, aged 74*
Provide more information	I: Because, even if you were going to have dialysis, you would like to receive a timeline? P: Yeah, rght, a timeline. For example, how long can I go on receiving dialysis until they say, “Well sir, your kidneys are now so bad that you need a kidney transplant.” And I actually missed that point; you do read a lot about that of course. I: About transplantation? P: Yeah, kidney transplants. And later on I thought, why hasn’t that been mentioned? *- Male, aged 76*
Share responsibility in SDM	P: In itself, I am satisfied. Only, if they say I need to prepare for dialysis treatment, I’dsay, “What’s the best treatment for me?” Now I have to decide for myself what that is. Of itself, there’s nothing wrong with that, but they may say that won’t suit me. I: Would you like more help with that? That they then go and see what suits you best? P: Actually yes. In consultation with me, of course. I: Yes, exactly. That you are really a team. P: Yes. Now, as I see it, it’s like, “Well, you go ahead and choose.” *- Male, aged 74*
Ask more questions	P: Maybe she should ask me, “Yes, why?” Ask me more about my reasons. Ask me why I say I don’t want dialysis. Dig deeper, like, “Why do you say that? How many children do you have? You have children, but do you want to see your children grow up?” I: Yes, asking for the reason behind it. P: Yes, exactly. *- Female, aged 57*
Look at the patient more holistically during SDM	P: A tip for her is to look at the whole picture and ask yourself what else is wrong with this man. [Besides his kidney disease] Because of course, I only come here for the kidneys, but in principle, liver and heart and so on are all there too. *- Male, aged 66*

*Note. P patient, SO significant other, I interviewer, SDM Shared decision-making, HCP Healthcare professional*

**Table 4:** Questions, themes and HCPs’ quotes

Themes	Quotes
**What is important in SDM for HCPs?**	
SDM is a continuing process	N: But, you know, that really is the tricky part of this type of conversation. It’s not one conversation in which the decision is made. […]. Patients don’t have to choose at the end of this consultation. So, that is the actual context in which you assess or have to assess these conversations. […] You know, I am not having this conversation with the idea of ending with a decision having been made. An initial conversation with the patient is a bit of an inventory, testing, what have you heard, is it all clear, what are their thoughts. You then proceed from that point. *- Male, Nephrologist*
Patients being aware of all options and risks	N: Yes, I think I am making an important decision here, in which I do try to take her along. But that’s difficult. If you, as a patient, have a completely different picture of what a treatment is and what the consequences are, then you first have to completely update that patient about that. You know, “What does that conservative therapy actually mean to you, it does mean that you die much earlier, do you actually realize that?” Otherwise, you will make the wrong decision. And the great thing about these conversations is that it doesn’t have to be decided right away. […] So here we have laid an important foundation on which to move forward. *- Female, Nephrologist*
Explore and validate patient’s motivation for options	N2: The key thing in such conversations is figuring out why is this patient choosing this kidney replacement therapy, what are the reasons, and why not choose the other? And to see if the patient’s arguments are correct. And whether the patient has a divergent image that is not correct that I may have to adjust. N1: Right. And I also think that the question you asked earlier – “What would you see as the best option?” – well, if patients choose that option, then that’s great. But if they choose something else, then you ask why. Why is this patient now choosing this option? *- Female and male, Specialist Nephrology Nurse and Nephrologist*
Take control in SDM	N: So I sometimes think the questions you ask the patient have to be in depth to understand whether the patient got it. But at a certain point, it has to stop – in the sense that you don’t inadvertently project your own insecurity onto the patient, which also makes them insecure. [...] You try to push off your uncertainty, letting the patient decide. I’m not that kind of doctor. I think that if I can and must decide something, I will. But that’s also because I’m the expert. Then a patient can also rest assured that I am taking the lead. […] When it comes to complicated medical-technical matters, I think we as doctors should be in the lead. *- Male, Nephrologist*
**What contextual factors influence SDM?**	
Knowing the patient	N: Yes, I spoke to him [the patient] at length afterwards, and also called him later on. So I think we are now getting to know this gentleman a little bit better and also understanding better what suits him and which stage he was in. Because he came here, a little unaware and uninformed, whereas he’s the kind of guy who wants to be in control, and I did not realize that at the time. So I think we are able to connect to him better now. *- Female and male, Specialized Nurse in Nephrology and Nephrologist*
General characteristics of the patient	N: Yeah, I find people [before starting treatment] who don’t want to hear anything about dialysis at all, and we have them too. […] They really don’t want to hear about it, and only want to talk about it “when the time comes”. […] You don’t want anyone to instantly start a treatment they know nothing about, especially if you can already see it coming. *- Female, Nephrologist*
Time available before consultation and start treatment	N: So, I like to take time for a conversation. But sometimes we have consultations, with an average of 10 minutes per patient, and then there’s that continuous pressure of a full waiting room. People always appreciate it, though, when you give them time, which I actually always do when necessary. *- Male, Nephrologist*
Differences between patient and HCP	N: And with her, she didn’t want something completely different than what I had thought of, or what could be suitable for her. I: And if it did, what would that have changed? N: Well, if she was someone who had a very strong treatment desire, where I would think that it doesn’t seem sensible on medical grounds, those are difficult conversations. *- Female, Nephrologist*
The organization of care in CKD	N: That makes it easier, having patients who know what they’re talking about. He also has been educated here about the different forms of dialysis. That’s the way we do it here: we have a certain way of educating, putting the doctor at the back of the process. And that has also helped and worked here. *- Male, Nephrologist*
**What role do HCPs see for themselves in SDM?**	
As team player	N: But I think that as a nephrologist, you can also do a lot in this [providing information about conservative treatment], but also as a renal failure team, as a dietitian, social worker, nurses … they all play important roles in this. *- Female, Nephrologist*
As information provider and advisor	N: Well, especially in the beginning, before they actually start treatment, I already start giving information. And my experience is that people often also need it. For some, the clearance [eGFR] is not that bad at all, but then questions arise. And then I usually refer them to the website, to read more information about it first. Because when people enter care for advanced chronic kidney disease, the clock’s already ticking. *- Female, Nephrologist*
Eliciting, checking and adjusting information	N: This is also something I often talk about with patients [the course of kidney failure], because in my experience, people just don’t really know what happens. And just like the patient, I also had a cat who died of kidney failure [the patient thought she would experience the same course of kidney failure as her cat], and I thought I had to adjust that image, because yes, she probably will not die like a cat. I was afraid she had all kinds of weird images in her head. […] I think she really had to know this, just how it goes. *- Female, Nephrologist*
As coach being supportive	N: Yes, I think that I am coaching and acting as a sounding board for this gentleman, I think, in the sense that he himself indicates what wishes and limits are, and I can also indicate what our wishes are and state the limits of what is and is not possible. It’s also to find out how he actually sees life and what he thinks about it, what role he has in it and what form suits it best. So, I actually try to think along with him to find a solution. […] So it’s mainly about hearing from him how he sees things, and returning it to him. He’s the kind of guy who can decide for himself, but who does need me as a sounding board. *- Female, Nephrologist*
As a practitioner taking responsibility	N: Yes, here I was being persuasive again because it appears that there is something that he hasn’t fully understood. And then I take on my role as a doctor and try to explain how it works. I: But what exactly is persuasion? N: Well, not so much persuasion, that’s not the right word, but that I explain the possibilities. So, I rise above the conservation, and explain, “No, but, you didn’t quite understand this”: I‘m taking on a bit of a leading role in the conversation again. […] But, that’s why I’m here, that’s my job, and that’s not negative. *- Female, Nephrologist*
**What can HCPs themselves do better in SDM?**	
Let the patient talk (provide teach-back)	N: Look, on the one hand, I always really love to wrap up a conversation with some sort of summary and conclusion, also to check if we both have the same idea of what we have agreed on. Well, I did here [referring to the fragment from the video-recorded consultation], and on the one hand, you can also say that it was persuasive and I should have let the patient summarize what we decided. *- Female, Nephrologist*
Ask the patient more questions	N: Well, he didn’t talk much and that gives me the feeling I was maybe talking too much [after looking at a fragment from the video-recorded consultation] […] Should I ask “What do you think about that?” more often. So, in between, give more back to that patient? Like, “We’ve now discussed this: is this clear to you?” That could perhaps be better – recapitulating every now and then. *- Female, Specialist Nephrology Nurse*
Be clearer to the patient and take control	N1: Still, I should maintain control and provide structure in the conversation. Right from the beginning of the conversation too, saying “This is going too far for now; let’s discuss the basics first and then you can talk again with the nurse.” We should mention that earlier. N2: Yes, maybe I should have said quite early in the conversation that we’d noticed they weren’t well-enough informed yet and we have to go back to providing information. *- Female and male, Specialist Nephrology Nurse and Nephrologist*
Persuasion in information provision	N1: I think – not so much in this conversation [referring to the video-recorded conversation] but rather in the process before that – that we could be a bit firmer. We [N1 & N2] already feel that PD would be a great option for this patient, therefore it is very important how you organize the provision of information about PD for him. N2: […] If we determine that someone should be informed, then we have a form for the nurse conducting the provision of information, in which the nephrologist’s preference for kidney replacement therapy is indicated. In some cases, the PD education comes first and the PD education is more extensive than the HD education, being a bit persuasive. *- Female, Nephrologist in training*
Taking time for SDM	N: This is something you generally do over a longer period [the SDM process]. Sometimes you don’t, because you don’t have a lot of time, caused by rapidly deteriorating kidney function. But if you have time, then you have the time to talk about that [about options for kidney replacement therapy], so I think you should take that time. On the other hand, you shouldn’t keep dawdling. […] So we will get through, we will continue, a decision will come, and it will be taken together in the foreseeable future, without us keeping running in circles. *- Female, Nephrologist*

Note *N* nephrologist or nurse, *I* interviewer, *SDM* Shared decision-making, *HCP* Healthcare professional, *CKD* Chronic Kidney Disease

### Patients’ perspectives

#### What is important in SDM for patients?

Most patients value the HCP’s *opinion* about suitable *medical options* because they believe that the HCP has more knowledge (Table 3). Nevertheless, the degree of significance assigned to the opinion of the HCP differs between patients. Some patients believe that the opinion of the HCP is very important for making a decision and that opposing that opinion is useless. Other patients perceive the HCP’s opinion as non-mandatory advice. These patients underline that the final decision still has to be made by them, not by the HCP.

The *type of information provision* in SDM is also deemed important by patients; however, patients differ in this respect. As illustrated in Table 3, some patients appreciate visiting, meeting and talking to other patients who are treated with dialysis, which supports them to conceive this particular treatment option. Other patients, however, strongly oppose meeting other dialysis patients as this would negatively affect their perception.

#### What can patients themselves do better in SDM?

Taking control and asking more questions were mentioned as *strategies* by patients to acquire more knowledge about the different treatment options. Another strategy that might work is taking notes during the medical visit.

#### What can HCPs do better in SDM according to patients?

All patients want their HCP to be *consistent and reliable* in the information that is provided during the SDM process (Table 4)*.* Furthermore, according to some patients, errors in scheduling hospital appointments arouse distrust, whereas HCPs’ attentiveness and availability is much appreciated and provides patients with a sense of security.

HCPs should *provide more information* and *ask the patients more questions* about their treatment preferences. In addition to getting a time indication for dialysis, and transplantation in general (Table 3), patients wanted more information about who is to decide if the patients remain in doubt, the prospects and prognosis of having limited kidney function, the causes of kidney failure (e.g. an unhealthy lifestyle), conservative treatment, the burden of dialysis on everyday life and on significant others.

### Healthcare professionals’ perspectives

#### What is important in SDM for HCPs?

According to most HCPs, SDM is *a continuing process* (Table 4). Most HCPs state that when a decision is made, this does not mean that this choice is irreversible. Some HCPs furthermore appreciate the input from significant others. After all, most patients will make their decision together with those significant others before visiting the hospital. In the process of SDM, HCPs see themselves only as confirming and documenting the final decision. Furthermore, HCPs vary in the significance they attach to decisions that have to be made, e.g. from a medical perspective, the choice for transplantation or conservative treatment is considered more important than the choice between haemodialysis (HD) or peritoneal dialysis (PD). Yet they do underline that this might not be the case for patients, for whom practical and everyday consequences of these treatment options are equally important.

Many HCPs said that patients *need to be aware of all options and risks.* Furthermore, according to the HCPs, it is important to *explore and validate* the motivation of patients for certain treatment options (Table 4). Many HCPs state that when patients have understandable, valid and medically realistic reasons, the decision is acceptable to them, even when that decision differs from their own preference. When patients’ reasons are not valid or based on incorrect arguments or not medically realistic, this expectation needs to be corrected by the HCP and the decision should be reconsidered.

HCPs indicated varying degrees of *taking control* during SDM. Many HCPs stated that taking control as medical experts and when medical technicalities are important (Table 4). However, within this role, some HCPs say that it is important to keep listening to patients’ preferences. For instance, when the cognitive abilities of patients are limited, HCPs indicated to take responsibility and control over the decisions of patients, and within this role, continuously try and elicit the preferences of patients. Some of the HCPs mentioned strategies that facilitate taking control during SDM: referring the patient to the transplantation-team and using the team’s information provision and advice to back the decision for deciding on a transplant; using the patient’s age as an argument for deciding on a transplant; tailoring information arguments with knowledge and information to persuade the patient towards a decision.

#### What are contextual factors that influence SDM according to HCPs?

According to some HCPs, not every patient wants to hear or talk about dialysis (Table 4). *Patient characteristics* mentioned by the HCPs that diminish patients’ active participation in decision-making are their psychological condition (e.g. distressed or depressed patients); their attitude (e.g. patients being rigid in their arguments and beliefs); their cognitive processes and abilities that are sometimes hard to follow.

Furthermore, HCPs experience differences between patients’ preferred treatment option and the medical possibilities (Table 4). Another aspect mentioned by HCPs as influencing SDM is when patients and HCPs differ in their expectations of the consultations. Also, according to most HCPs, differences in medical knowledge and expertise influence SDM.

#### What role do HCPs give themselves in SDM?

In general, HCPs see five roles for themselves in SDM (Table 4). When patients are cognitively too impaired to decide, HCPs’ role is to *coach and support* the patient. In this role, HCPs emphasize being explorative, empathic and comforting to the patient. However, when patients are in doubt, their role of a *responsible practitioner* with a more rigid, directive and persuasive communication style prevails. Some HCPs said that, from a medical perspective, the HCP should always take the lead, taking responsibility for accurate information provision and SDM in general.

#### What can HCPs themselves do better in SDM?

In addition to maintaining control and providing structure in the conversation, other ways to be *more clear towards the patient and take control* were summarizing more often, using fewer words, refraining from too much in-depth conversation, being more clear and honest about the possibility for transplantation and the end-stages of conservative treatment.

## Discussion

This explanatory study assessed the extent to which SDM was accomplished during consultations in nephrology in which a final decision for kidney replacement therapy has to be made. Overall, SDM is applied by the HCPs to a moderate extent. Compared to SDM scores in Dutch oncological and palliative care settings [31, 32], the average SDM score in Dutch nephrology seems promising. However, improvement is still needed, given recent survey results indicating that 30% of patients with kidney failure say they did not receive information or support when choosing a kidney replacement therapy [11]. Our interviews showed that all patients rely on the information provision and opinions of HCPs during SDM, expect consistency and support, and want a proactive role in SDM. They would like to receive more information from their HCPs and to be questioned about their preferences. HCPs appear willing to incorporate patients’ preferences in SDM when there are no medical contraindicators, the patient is fully informed and decides using valid arguments. HCPs also like the patient to take a more prominent role in SDM which is in accordance with previous research [33].

Our quantitative observations showed that HCPs least often reassure patients or reaffirm that they will support them by giving information or deliberating the options (2nd item of the OPTION instrument). This element of SDM seems particularly important in nephrology, though, given the difficulties patients face in choosing a preference-sensitive treatment option [2–4]. In addition, patients with limited health literacy, i.e. a limited ability to access, understand, appraise and apply health information in making healthcare decisions [34], are prevalent in kidney care [35]. These patients are known to be relatively passive during SDM, less inclined to take control during the conversation and prone to follow the HCP’s advice [36, 37]. For them, additional support to let them become better informed or deliberate options seems needed.

Supporting patients in their decision-making process should not be restricted to the interaction during scheduled outpatient visits. A patient who has received information about the various treatment options beforehand will participate more actively in the decision-making process with the HCP. To that end, various tools have been developed in the Netherlands as well as in other countries. An example of this is the decision aid on kidney failure (in Dutch only) developed in close collaboration with nephrologists. In addition, the Dutch patient organization for patients with kidney disease developed an educational website (Nierwijzer.nl) with specific information about the various treatment options [1]. For patients with lower levels of health literacy and known to have difficulty reading and understanding written language, educational tools are available with visual information and illustrations that help them understand better what the different options entail. These tools can also be discussed during outpatient visits. However, discussing treatment options and participating in decision-making requires more than being informed. As our study shows, specific communication skills are needed such as asking questions, expressing concerns and interrupting an HCP when the information provided is too complex or upsetting. Patients often experience communication barriers, as was also demonstrated in a previous study among patients with different types of chronic diseases [38]. Designing communication-enhancing interventions together with patients could help them to overcome these barriers. Such interventions can, however, only be effective when HCPs also take their communicative role seriously and realize that inquiring about patients’ treatment preferences and listening to their needs and worries is crucial for achieving the goal of person-centred care and preventing the implicit persuasion which now occurs quite often and hinders patient participation in decision-making [11].

An important strength of this study is that we assessed SDM in everyday practice in multiple centres and discussed patients’ and HCPs’ contributions to this process afterwards. Furthermore, real-life video-recorded consultations were collected, representing valid and reliable data. In addition, the stimulated recall interviews were perceived as educational and worth the invested time. A final strong point was that various elements of the communication were unravelled in this study, giving nephrologists an understanding of many educational aspects of SDM.

Nevertheless, there are also some methodological weaknesses. First of all, the inter-rater reliability between the two observers was only moderate and the average SDM score was somewhat higher according to the second observer. This could point to an underestimation of SDM. However, the main coder had more experience in nephrology and therefore the (lower) coding by the main coder is represented in the results section. Even so, both observers were trained and have ample experience in coding with the OPTION. Second, in addition to how it is applied by HCPs, SDM also depends on patient characteristics and the context of the consultation [39]. Considering this, the relatively small number of hospitals (*n* = 7) and consultations (*n* = 29) and the explorative research design could have reduced the study’s external validity. Additionally, because of the explorative design and relatively small number of participants available, data saturation in the qualitative part of this study is also difficult to discern. Still, within the clinical context of strict privacy regulations and time constraints, 14 nephrologists allowed us to make recordings of their consultations and participated in the stimulated recall interviews. Thirdly, one consultation was recorded and analysed per patient. However, as HCPs’ interviews showed, SDM is a continuing process that involves multiple consultations with multiple HCPs. Information not discussed in the observed consultation could therefore have been mentioned in earlier consultations, indicating a possible underestimation of SDM. Fourthly, 15 HCPs participated in this study. They were the ones who agreed to participate out of a group of 28 HCPs who were invited to join. We did not record the characteristics of the non-responders. It is perfectly possible that they are not representative of the overall group of nephrologists in the Netherlands: maybe more skilled communicators volunteered, maybe less-skilled ones did. Our SDM ratings could therefore be an overestimation, although we did find similar ratings in other medical specialties [31, 32]. Lastly, the OPTION^5^ instrument does not measure the overall quality of communication [17]. This could mean that although SDM scores are moderate, the overall quality of communication is still adequate. For a more conclusive and comprehensive assessment of communication and SDM, other communication and decision-making aspects should be measured as well. An example of this is (implicit) persuasion, in which HCPs, despite their best intentions, convey a treatment preference and steer the decision [11]. Persuasion is mentioned by HCPs in our interviews when describing their role in SDM.

## Conclusions

In conclusion, in Dutch nephrology, SDM is applied by HCPs to a moderate extent. However, every patient should be involved in SDM. In the case of kidney failure, encouraging patient participation is even more important, given the complexity of the disease, the relatively high prevalence of limited health literacy in patients [40] and the life-changing choices they are required to make. Reductions in SDM could result in decisional regret [41], harm the quality of life [42, 43] and lead to (unwarranted) practice variation [44]. Offering patients tools to support decision-making, such as educational websites and decision aids, could prevent these unwanted outcomes.

## Supplementary Information

**Additional file 1.**

## Data Availability

The datasets (video recordings of outpatient visits) generated and analysed during the current study are not publicly available due to the highly private nature of the recordings but are accessible through the corresponding author on reasonable request.

## Abbreviations

SDM: Shared decision-making
HCP: Healthcare professionals (in nephrology)
CKD: Chronic kidney disease
