# Supplementary material for: Practices and perspectives of patients and healthcare professionals on shared decision-making in nephrology
Source: BMC Nephrol. 2022 Jul 21;23:258. doi: 10.1186/s12882-022-02887-4 (PMC9306155; doi:10.1186/s12882-022-02887-4)
Supplement: Supplementary file 1 — Additional file 1. [file 12882_2022_2887_MOESM1_ESM.pdf]

Appendix 1 Topic list for patients

In the study "DIALOOG" (Dutch for "Dialogue"), we would like to investigate how the HCP talks to you about the treatment you need. We do this by making a video recording of a conversation with the HCP. From this, we choose short video fragments that we can review and discuss with you within a month. This interview will last approximately 45 minutes. An audio recording is made of this conversation and is typed out by the researcher.

General questions at the beginning [before watching video fragments]

1. Do you remember the conversation we recorded? The conversation is recorded on [INSERT DATE VIDEO-RECORDED CONSULTATION] with HCP [INSERT NAME HCP].

2. How stressed are you at the moment? (VAS)

   Not at all Very

   ●---●

   0 10

3. How satisfied are you with the way the HCP communicated with you? (VAS)

   Not at all Very

   ●---●

   0 10

4. To what extent did the HCP sympathize with you during the conversation? (VAS)

   Not at all Very

   ●--●

   0 10

5. Why did you go to the HCP at that moment?

6. What did the HCP discuss with you at that moment?

    a. For example, which treatment options, including the option not to treat?

7. During the visit, have you made a treatment decision?

    a. What did you choose at that moment? And why?

    b. Who decided on treatment?

    c. Was it clear to you what you needed to decide on?

    d. Did you feel supported by the HCP in making this decision?

    e. Did the HCP provided you with enough information about the treatments (advantages/disadvantages of each treatment/choice)?

    f. Were you able to express your wishes to your HCP?

    g. Did the HCP take your wishes into account?

    h. Did you feel informed enough to decide?

    i. Do you still support the treatment decision you made at the time (decisional regret)?

    j. Have you already started treatment?

8. Did the HCP sufficiently ask about your problems or complaints?

9. Were you able to talk about your concerns or fears?

10. Is the relationship with your HCP on the basis of equality?

11. Have you been able to ask the questions you wanted to ask?

12. Did the HCP use difficult words that you did not understand? If so, can you tell us a bit more about that? [If necessary, ask the patient to "point this out" in one of the fragments].

Questions directly after watching each video fragment

1. How would you grade this part of the conversation (0-10)? Why?

2. What do you think about the way you talked here?

3. What do you think about the way the HCP talked with you here?

4. What did you find difficult in this fragment?

    a. For example, particular words the HCP used, or the fact that the HCP told many different things.

5. What did you like about this fragment?

6. Did you feel like the HCP gave you enough room?

    a. to talk

    b. to ask questions

    c. if in doubt

    d. if you disagreed with something

    e. if you didn't understand something

7. Did the HCP and you decide together? Why / why not?

8. Did you feel like the HCP preferred a particular treatment?

    a. And what is your opinion on that? Did you agree?

9. Did you feel like you were taken seriously by your HCP in this fragment?

10. How did you emotionally feel during this part of the conversation?

11. Are there things you would do differently next time? (e.g., asking more questions or prepare yourself better)?

12. Would you like your HCP to do things differently next time?

13. Do you have anything else to add?

General questions at the end [after watching video fragments]

1. How did it make you feel to watch your own visit?

2. How did you feel experience answering questions about your visit?

3. Do you have anything else to add?

Appendix 2 Topic list for HCPs

In the study "DIALOOG" (meaning "Dialogue"), we investigate how treatment options for kidney failure are discussed, and how decisions are made. We do this by recording consultations on video and selecting video fragments that we discuss with the HCP in a "stimulated recall session". In this recall session, the HCP and researcher (without the patient) reflect on an average of three fragments from video-recorded consultation. This will take approximately 45 minutes. Consent for this recall session and the audio-recording of the session is already included in the consent form for permitting the video-recording of the consultation. After transcribing, the audio recording will be deleted.

General questions at the beginning (before watching video fragments)

1. Do you remember the conversation we recorded? I'm referring to the conversation recorded on [INSERT DATE VIDEO-RECORDED CONSULTATION] with a [INSERT GENDER PATIENT] aged [INSERT AGE PATIENT] with the name [INSERT NAME PATIENT].
2. What did you think of this conversation? How did you feel about that?
3. Which (contextual) factors (e.g., time pressure, emotions of the patient, comprehension or language problems) played a role in the way this conversation proceeded?
4. Would you describe the relationship with this patient as equivalent?
5. Decision-making:
    - a. What treatment options does this patient have?
    - b. Which advantages and disadvantages of these treatment options do you consider relevant for this patient? Why do you think so?

c. Which treatment (or no treatment) options do you think the patient should choose? Why do you think so?

d. What do you think, did the patient and you make a shared decision during the consultation?

e. How would you describe your role in decision-making?

i. Are you satisfied with this?

ii. What would you like to do differently in future consultations?

f. During the consultation, did you provide the patient with sufficient information about the treatment options so that the patient can decide himself? Could you perhaps elaborate on this using an example?

g. Do you think the patient has understood the information and the consequences of his or her choice?

6. Do you think this patient felt supported during the conversation?

a. Why / why not?

7. Do you think this patient feels taken seriously?

a. Why / why not?

8. Were you able to discuss everything you wanted with this patient?

a. If not, why not?

b. If you weren't able to discuss everything, what does this mean for the decision-making process?

Questions directly after watching each video fragment

1. How would you grade this part of the conversation focused on communication (0-10)? Why?

2. What did you think of the way you talked to the patient in this fragment (not substantively, but communication-oriented)?

3. What did you think of the way the patient talked to you in this fragment (not substantively, but communication-oriented)?

4. If applicable: What do you think: did the patient and you decide together?

    a. Why / why not?

5. If applicable: What do you think of the way you provide information to the patient in this fragment?

    a. To what extent do you think you are neutral in your information provision?

    b. Do you and this patient in this fragment have an equivalent interaction with each other? Why do you think so?

6. If applicable (if a decision is made in the fragment): How would you describe your role in SDM during this fragment?

    a. What is your opinion on this? What role do you prefer?

7. How did you feel during this part of the conversation?

8. Do you have anything else to add to this?

General questions at the end (after watching video fragments)

1. When thinking about the conversation as a whole, what were difficult moments? (these can also include other moments than the previously discussed fragments).
2. How satisfied are you with your communication?

Not at all satisfied — Very satisfied

1 2 3 4 5 6 7 8 9 10

3. Empaty / stress.

   a) How much empathy have you shown during the conversation?

0% empaty ●————————● 100% empaty

   b) How emotionally stressful did you find the conversation?

0% stress ●————————● 100% stress

4. After reviewing the fragments, what would you like to change in your decision-making with patients in the future?

   a. Would you like to change your role during SDM?

5. Do you have anything else to add to this?
